# Storage of Fruits and Vegetables in Refrigerator Increases their Phenolic Acids but Decreases the Total Phenolics, Anthocyanins and Vitamin C with Subsequent Loss of their Antioxidant Capacity

**DOI:** 10.3390/antiox6030059

**Published:** 2017-07-24

**Authors:** Joseph H. Y. Galani, Jalpesh S. Patel, Nilesh J. Patel, Jayant G. Talati

**Affiliations:** 1 Department of Agriculture and Veterinary Medicine, Université des Montagnes, P.O. Box 208, Bangangté, Cameroon; 2 Department of Biochemistry, College of Basic Science and Humanities, Sardarkrushinagar Dantiwada Agricultural University, Palanpur-385001, Gujarat, India; jalpeshpatel.2729@yahoo.com; 3 Department of Biochemistry, B.A. College of Agriculture, Anand Agricultural University, Anand-388110, Gujarat, India; njp_19@yahoo.co.in (N.J.P.); jgtalati@yahoo.co.in (J.G.T.)

**Keywords:** fruits, vegetables, postharvest storage, phenolics, UPLC, antioxidants, principal component analysis

## Abstract

It is of paramount importance for consumers, scientists and industrialists to understand how low-temperature storage of food items affects their bioactive compounds and properties. This study evaluated the effects of cold storage on total phenolics (TP), phenolic acids profile (PA), total anthocyanins (TA), total ascorbic acid (Vit. C) and antioxidant activity (AA) of 19 fruits and vegetables, collected from local Indian markets and stored in refrigerator (4 °C) during 15 days. Content of TP was highest in dill and amaranth and decreased (up to 29.67%) with storage. Leafy vegetables (amaranth, dill, onion, fenugreek and spinach) contained higher amounts of the 12 PA revealed by UPLC-UV; ellagic, gallic, sinapic and vanillic acids levels were the highest; chlorogenic acid (ρ = 0.423), syringic acid (ρ = 0.403) and sinapic acid (ρ = 0.452) mostly correlated with TP; and the PA increased during storage. Highest contents of Vit C estimated by AOAC, DCPIP and DNP methods were found in amaranth, dill and pomegranate, and decreased with storage. Pomegranate showed highest TA levels and low-temperature storage did not significantly increase TA, which was the largest contributor of TP in fruits and vegetables (ρ = 0.661). Storage induced a drastic decrease of AA, which mostly correlated with TP (ρ = 0.808, 0.690 and 0.458 for DPPH, ABTS and FRAP assays, respectively). Spearman’s correlation confirmed by principal component analysis demonstrated that dill, pomegranate and amaranth had the highest overall antioxidant capacity, whereas orange juice and carrot showed the lowest. The results provide support for a key-role of TP, followed by Vit. C and TA in antioxidant capacity of fruits and vegetables, which could be interesting dietary sources of natural antioxidants for prevention of diseases caused by oxidative stress.

## 1. Introduction

Understanding the changes in phytonutrient content during postharvest handling of fruits and vegetables is important for both basic knowledge of postharvest physiology and for the food and health industries. It is also important for consumers and the larger audience to know the changes which occur in the nutritional quality and health-promoting compounds of the food they consume after storage in the household refrigerator. Beyond supplying energy and good quality proteins, fruits and vegetables are also significant sources of phytonutrients such as antioxidants, which possess health benefits. Antioxidants derived from plant sources may play an important role in reducing the risk of degenerative diseases such as cancer, obesity, cardiovascular disease and diabetes [1]. Bioactive secondary plant metabolites like flavonoids, carotenoids, and plant sterols, have been linked to health protection in in vitro, in vivo, and clinical research studies [2]. The phytochemical content of food items can be affected by postharvest handling procedures such as curing time, irradiation time, exposure to light, and temperature due to biochemical tissue responses [3]. Because many fruits and vegetables are highly perishable, after harvest, they are stored in cold conditions for many days before consumption. The effects of cold storage on fruits and vegetables have been previously investigated and varied considerably, depending on the species and the compounds under study.

Different patterns of variations in the total phenolic content (TP) and antioxidant activity (AA) of potatoes during low-temperature storage were reported [4,5,6,7,8]. Rapisarda et al. [9] observed during storage at 6 °C for 65 days, an increase in anthocyanins, flavanones and hydroxycinnamic acids and a decrease in vitamin C in the blood oranges, with an increase in antioxidant capacity. A rapid decrease in total AA, ascorbic acid and total phenolic compounds concentration was observed in broccoli stored at 1 °C for 28 days [10]. Piljac-Žegarac and Šamec [11] reported that small fruits like strawberries, raspberries, red currants, cherries, and sour cherries stored at 4 °C, exhibited slightly higher AA values as compared to the storage at 25 °C. Moreover, correlations between AA and phenolic components in different fruits and vegetables were established [3,7,8,9,10,11,12].

During storage of fruits and vegetables in cold condition, it was observed that ascorbic acid or Vit. C content decreased [7,9,13,14,15,16,17], but Phillips et al. [15] reported a stability of Vit. C in clementine and orange juice. Vitamin C contents in fruits and vegetables show great variations and also depend on the extraction solvent and extraction method, as well as the method of quantification [18,19,20,21]. Hence, it is important to know which changes (increase or decrease) and the degree of change induced by storage of fruits and vegetables in refrigerator. On the other hand, the influence of vitamin C on the antioxidant capacity of fruits or vegetables is not clear [22,23]. In this study, we have estimated Vit. C losses in fruits and vegetables using one titrimetric method (2,6-dichlorophenol-indophenol-DCPIP or AOAC method), and two spectrophotometric methods (DCPIP spectrophotometric method and 2,4-dinitrophenylhydrazine-DNP method).

Antioxidant databases of a wide range of vegetables and fruit would be useful for dietary guidelines and epidemiological research [24]. However, earlier researches focused mainly on a few fruits or vegetables separately, whereas an experiment including a considerable number of both the commodities would provide a better idea of the phytochemical changes happening during cold storage. Besides, although India is among the larger producers of fruits and vegetables, very limited information is available on the bioactive content and antioxidant properties of Indian cultivars commonly found on the market. Considering the large vegetarian Indian population whose diet is rich in fruits and vegetables, these data are much needed. Each antioxidant has varying contributions to the total antioxidant potential in different methods. So, for evaluation of antioxidant capacity, the use of several methods at a time is recommended to obtain a comprehensive view of the antioxidant potential of each biological sample [25]. Consequently, in this work, to determine the antioxidant capacity of fruits during storage, we used 2,2-diphenyl-1-picrylhydrazyl (DPPH) free radical-scavenging ability, 2,2′-azinobis (3-ethylbenzthiazoline-6-sulfonic acid) (ABTS) radical cation decolorization assay, and ferric reducing antioxidant power (FRAP) assay.

On the other hand, only cursory attention has been given to changes in individual phenolic acids in these food items and their contribution to the AA. Therefore, it is important to understand how cold storage affects the phytonutrient content and bioactive properties of Indian fruits and vegetables and determine the precise influence of each phytochemical compound on bioactive properties, for instance AA. This paper examines how storage in the refrigerator affects the TP, PA composition, TA and Vit. C contents of 19 fruits and vegetables grown in India, with subsequent impact on their AA, and it investigates which compounds influence the AA of these food items.

## 2. Materials and Methods

### 2.1. Chemicals

Solvents for extraction and reagents for colorimetric tests of analytical grade were procured from Himedia Laboratories (Mumbai, India). For UPLC profiling, HPLC grade phenolic acid standards (gallic acid, chlorogenic acid, caffeic acid, vanillic acid, syringic acid, para-coumaric acid, ferulic acid, sinapic acid, salicylic acid, ellagic acid and trans-cinnamic acid) were purchased from Sigma-Aldrich (St. Louis, MO, USA), protocatechuic acid was procured from HWI Analytik GmbH (Ruelzheim, Germany). Solvents for UPLC separation (HPLC and LC–MS grade) were obtained from Merck Specialties (Mumbai, India).

### 2.2. Plant Materials and Treatment

All 19 food items were purchased from a local market at Anand, Gujarat, India. They included six fruits *viz*., apple (*Malus domestica*), banana (*Musa* sp.), grape (*Vitis vinifera*), orange (*Citrus sinensis*), pomegranate (*Punica granatum*) and sapota (*Achras zapota*), and 13 vegetables, among which five were green leafy vegetables *viz*., amaranth leaves (*Amaranthus hypochondriacus*), dill leaves (*Anethum graveolens*), fenugreek leaves (*Trigonella foenum-graecum*), onion leaves (*Allium cepa*) and spinach leaves (*Spinacia oleracea*), and eight were other vegetables i.e., cabbage (*Brassica oleracea* L. var. capitata), carrot (*Daucus carota* subs. sativus), cauliflower (*Brassica oleracea* L. var. botrytis), eggplant (*Solanum melongena*), green pepper (*Capsicum annum*), potato (*Solanum tuberosum* var. Kufri Lauvkar), sugar beet (*Beta vulgaris*) and tomato (*Solanum lycopersicum*). The food items were randomly separated in two batches: the first batch was immediately processed in the laboratory and the second one was stored in refrigerator at 4 °C during 15 days. For each sampling period, five pieces of each commodity were randomly taken, thoroughly washed with tap water and dried with blotting paper. Then the edible portion was cut into small pieces and pooled. The pooled sample was powdered with liquid nitrogen using a mortar and pestle, and if required, the freeze dried powder was stored at −70 °C until analysis. The oranges were squeezed by a domestic juicer, the juice was filtered, weighed and the volume of juice obtained was recorded and used to calculate the density to express the data on a fresh weight basis.

### 2.3. Extraction of Phenolic Compounds

The protocol was adapted from the method optimized by Jansen and Flamme [26]. One gram of freeze dried sample or 1 mL of orange juice was extracted in 6 mL of methanol/water/acetic acid (80:19:1, v/v/v), sonicated (Transsonic Digital sonicator, Elma, Singen, Germany) for 10 min at room temperature. The mixture was then centrifuged at 5000 rpm for 10 min (Laborzentrifugen 3K15 refrigerated centrifuge, Sigma, Osterode am Harz, Germany) and the supernatant was collected. The extraction of the pellet was repeated two more times, the two supernatants combined and the final volume made to 20 mL. The extracted phenolic compounds were stored at −20 °C for a maximum of 15 days before analysis.

### 2.4. Determination of Total Phenolic Content

The total phenolic content was determined according to a modified Folin-Ciocalteu colorimetric method. The sample solution (0.5 mL) was allowed to react with 3 mL of freshly diluted 10-fold Folin-Ciocalteu reagent, followed by the addition of 3 mL of sodium carbonate solution (60 g/L), and vortex mixed. After 90 min at room temperature, the absorbance reading was recorded at 725 nm (UV-1800 spectrophotometer, Shimadzu, Kyoto, Japan) against a blank of the extraction solvent. The standard curve was prepared using 20–100 mg/L solutions of gallic acid in the extraction solvent. The contents of TP were expressed as milligrams of gallic acid equivalent (GEA) per 100 g of fresh weight of the edible portion (mg GAE/100 g fw).

### 2.5. Profiling of Phenolic Acids by Ultra Performance Liquid Chromatography (UPLC)

The composition of PA was analyzed from TP extracts by UPLC-UV as described by Galani et al. [8]. A Waters Acquity UPLC^TM^ H Class System (Waters Corp., Milford, MA, USA) equipped with a Quaternary Solvent Manager and a Sample Manager FTN was coupled to a UV photodiode array detector (PDA) was used. The separation was performed on a bridged ethylene hybrid (BEH) C18 analytical column (1.7 µm, 2.1 mm × 50 mm, Waters Corp.). Calibration curves for the standards were obtained at concentrations ranging from 10 to 100 µg/g and the content of each PA was calculated against its standard curve and expressed in µg/100 g fw.

### 2.6. Determination of Vitamin C Content

Vitamin C was extracted from 0.5 g of freeze dried samples or 1 mL of orange juice in 4 mL of 5% w/v aqueous solution of metaphosphoric acid containing 1% w/v dithiothreitol (DTT) following the protocol described by Külen et al. [7] and estimated using 3 different methods: the DCPIP titrimetric or AOAC method [21], DCPIP spectrophotometric method [19] and the modified DNP method reported by Cho et al. [17]. The Vit. C content was expressed in mg/100 g fw.

### 2.7. Determination of Total Anthocyanins

The method was adapted from Burgos et al. [26]: 0.2 g of freeze dried samples or 1 mL of orange juice was mixed with 10 mL of methanol/1.0 M HCl (75:25, v/v) and sonicated for 10 min at room temperature. The mixture was centrifuged at 5000 rpm for 10 min and the pellet was re-extracted. The combined supernatants were filtered and the volume made up to 25 mL with the extraction solution. Absorbance of the extract was read at 545, 535 and 515 nm and the concentration of TA was calculated using the molar extinction coefficient and molecular weight of malvidin-3-*p*-coumaroyl-glucoside for blue-violet pigments (545 nm, 3.02 × 10^4^ L/mol/cm, 718.5 g/mol), pelargonidin-3-glucoside for red pigments (515 nm, 2.73 × 10^4^ L/mol/cm, 486.5 g/mol) [27], and cyanidin-3-glucoside for purple pigments (535 nm, 3.43 × 10^4^ L/mol/cm, 449.2 g/mol) [28]. Results were expressed in mg/100 g fw.

### 2.8. Evaluation of Total Antioxidant Activity

Antioxidant activity of the TP methanol extracts was evaluated using modified DPPH and ABTS assays adapted from Madiwale et al. [6] with 6-hydroxy-2,5,7,8-tetramethylchroman-2-carboxylic acid (Trolox) as a standard, and expressed as mg of Trolox equivalents per 100 g of fresh sample (mg TE/100 g fw). The FRAP assay was performed according to Kubow et al. [29] and expressed in µmol Fe(II)/100 g fw.

### 2.9. Statistical Analysis

All experiments were performed in triplicate. Data obtained were analyzed using Analysis of Variance (ANOVA) carried out at 0.05 level of significance to determine the significance of difference before and after storage of food items. For each fruit or vegetable, differences between treatments were determined by Fisher’s Least Significant Difference at 0.05. As the data was not normally distributed, the relationship between phytochemical contents and antioxidant parameters was characterized by calculating the Spearman’s correlation coefficients [30]. A principal component analysis (PCA) was applied as described by Patras et al. [31] to illustrate a biplot representing how storage in cold conditions influenced each parameter in each food item. The statistical analyses were performed using XLSTAT version 2017.2 software (Addinsoft, New York, NY, USA).

## 3. Results

### 3.1. Total Phenolics

**Figure 1:**
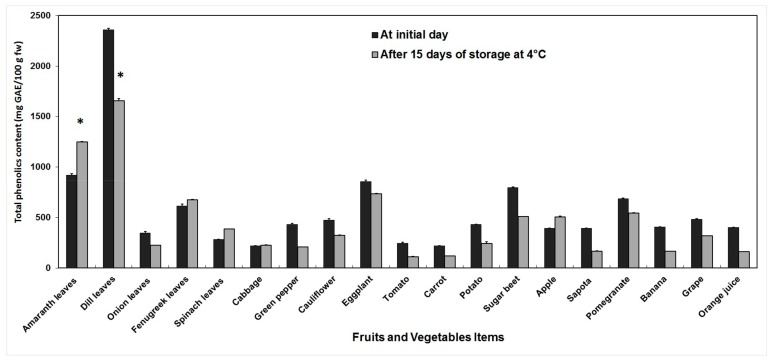
Changes in total phenolic content of fruits and vegetables before and after 15 days of storage at 4 °C. Total phenolics were extracted in methanol and measured by Folin-Ciocalteu colorimetric method. Error bars represent standard error of means of three replicates. * indicate significant differences (Fisher’s LSD, *p* < 0.05) between values before and after storage.

The content of TP varied between 216.97 and 2356.96 mg GAE/100 g fw before storage, and between 112.43 and 1657.69 mg GAE/100 g fw after storage. Dill and amaranth showed higher amounts as compared to other food items, while the lowest TP values were obtained in tomato and carrots. In general, storage of fruits and vegetables at 4 °C during 15 days significantly decreased their TP content, with the highest significant TP loss (29.67%) recorded in dill, although an increase of TP was observed in amaranth, fenugreek, spinach, cabbage and apple, with up to 35.81% increase observed in amaranth (Figure 1).

### 3.2. Phenolic Acids

**Figure 2:**
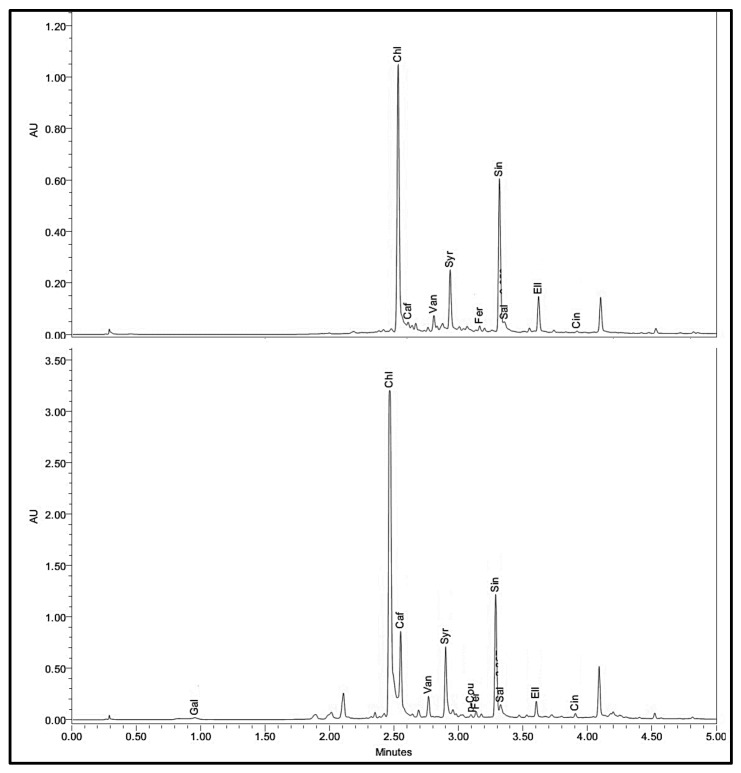
Sample UPLC chromatogram at 280 nm of dill leaves phenolic acids before and after 15 days of storage at 4 °C. Phenolics were extracted in methanol, filtered and 2 µL were separated through a BEH C18 column at 0.45 mL/min with 0.1% formic acid in water and 95% methanol in 0.1% formic acid. Gal = Gallic acid, Pro = protocatechuic acid, Chl = chlorogenic acid, Caf = caffeic acid, Van = vanillic acid, Syr = syringic acid, p-Cou = para-coumaric acid, Fer = ferulic acid, Sin = sinapic acid, Sal = salicylic acid, Ell = ellagic acid and Cin = *trans*-cinnamic acid.

The PA profiling results are summarized in Table 1. From the UPLC chromatograms, 12 PAs were identified and quantified: gallic acid (Gal), protocatechuic acid (Pro), chlorogenic acid (Chl), caffeic acid (Caf), vanillic acid (Van), syringic acid (Syr), para-coumaric acid (p-Cou), ferulic acid (Fer), sinapic acid (Sin), salicylic acid (Sal), ellagic acid (Ell) and *trans*-cinnamic acid (Cin) (Figure 2).

Initially, only amaranth contained gallic acid at 7960.53 mg/100 g fw. During storage, its content increased significantly to 18,886.11 mg/100 g fw, that is, an increase of 137.24%. Also, gallic acid, not detected before storage, could be found in dill, onion, cabbage, tomato, sugar beet and grape after storage. The highest content of protocatechuic acid (121.44 mg/100 g fw) was found in amaranth and it significantly increased to 335.09 mg/100 g fw during storage. No significant increase or decrease of protocatechuic acid content was observed in some fruits and vegetables, whereas the compound could not be detected in the others. Chlorogenic acid was mostly found in amaranth, dill and eggplant. During storage, a significant increase of 286.12%, from 236.23 to 912.15 mg/100 g fw was observed in dill, and no any significant increase or decrease was observed in the other food items. Dill and eggplant contained highest amounts of caffeic acid, followed by amaranth, onion, fenugreek and cauliflower. A significant and drastic increase from 1.16 to 57.72 mg/100 g fw was observed in dill during storage. High contents of vanillic acid were determined in dill, onion and fenugreek leaves, followed by amaranth, spinach, cabbage, cauliflower and carrot. During storage, significant increases of vanillic acid were observed in dill, onion and fenugreek leaves, with a 296.23% rise, from 432.64 to 1714.27 mg/100 g fw obtained in dill.

Syringic acid was mostly found in dill (17.25 to 52.31 mg/100 g fw) and fenugreek leaves (36.39 to 52.73 mg/100 g fw), before and after storage, respectively. Storage led to a significant increase of syringic acid content in the fruits and vegetables, with up to 203.25% increase in dill.

The content of *p*-coumaric acid ranged between 0 to 2.68 mg/100 g fw before storage, and 0 to 2.28 mg/100 g fw after storage. It was higher in amaranth, dill, fenugreek and spinach leaves, whereas considerable amount was found in other food items. During storage, significant increases were found in amaranth (110.45%), dill (100%); onion (108.82%) and spinach (1478.57%), meanwhile, significant losses were observed in fenugreek (60.45%) and orange (88.00%).

Ferulic acid was higher in dill, onion and fenugreek leaves, followed by amaranth, spinach cabbage and cauliflower which also contained substantial amounts. Storage a low temperature generated a significant increase of Fer levels in dill (from 1.76 to 5.61 mg/100 g fw), onion (from 1.01 to 7.60 mg/100 g fw), fenugreek (from 6.98 to 10.52 mg/100 g fw), and cabbage (from 0.42 to 2.01 mg/100 g fw).

Sinapic acid was found in all the food items, except banana. Dill, onion and fenugreek leaves showed the highest amounts, followed by amaranth, eggplant and spinach. During storage, a significant increase of sinapic acid (93.34%), from 2587.19 to 5001.95 mg/100 g fw was recorded in dill, the other food items showed no significant increase or decrease.

Except for banana and eggplant, salicylic acid was present in all the food items, with high levels in dill, onion, fenugreek and grapes. Storage enhance significantly the salicylic acid content in dill (from 7.97 to 42.21 mg/100 g fw), fenugreek (from 11.37 to 27.22 mg/100 g fw) and orange (from 1.06 to 7.24 mg/100 g fw) while a significant loss of 49.84% was observed in grapes (from 27.81 to 13.95 mg/100 g fw).

Dill showed the highest content of ellagic acid before and after storage, 12,231.48 and 12,429.06 mg/100 g fw, respectively. High amounts were also found in onion, spinach, cabbage and fenugreek. During storage, a significant increase in ellagic acid content (709.96%) was observed in spinach and no significant changes were recorded in the other food items.

Cinnamic acid was found in high amounts in amaranth (82.73 to 52.35 mg/100 g fw), dill (24.01 to 173.46 mg/100 g fw) and spinach leaves (48.24 to 306.80 mg/100 g fw), and moderate amounts were measured in onion, fenugreek, carrot and orange. Cold storage drastically elevated its content; significant increases of 622.45 and 535.99% were recorded in dill and spinach leaves, respectively.

In general, the PA found in high amounts were ellagic, gallic, sinapic and vanillic acids, while the lowest were caffeic, para-coumaric and syringic acids. Chlorogenic and sinapic acids were the most distributed PA among the fruits and vegetables, and gallic acid was the least distributed. The leafy vegetables (amaranth, dill, onion, fenugreek and spinach) contained higher amounts and most of the PA; among them, dill showed the highest increase of most of the PA during storage. Overall, storage increased the PA content of fruits and vegetables.

### 3.3. Vitamin C

**Figure 3:**
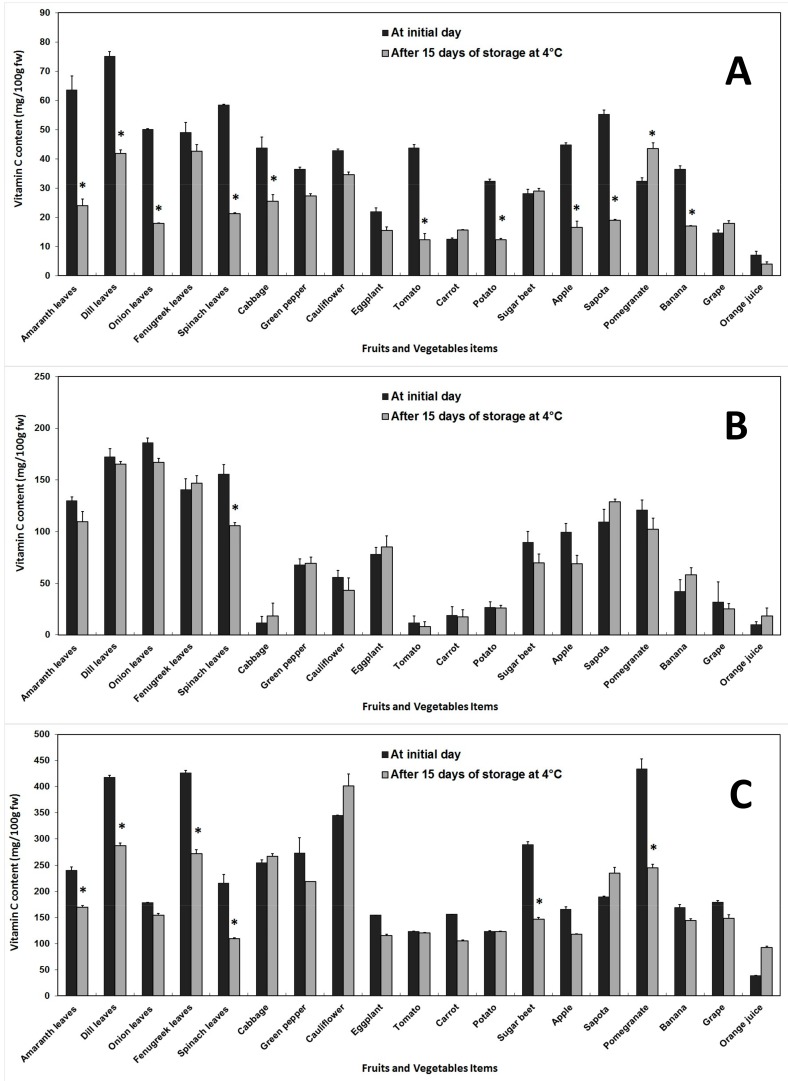
Changes in vitamin C content of fruits and vegetables before and after 15 days of storage at 4 °C, extracted in metaphosphoric acid and measured by AOAC method (**A**), DCPIP spectrophotometric method (**B**) and DNP method (**C**). Error bars represent standard error of means of three replicates. * indicate significant differences (Fisher’s LSD, *p* < 0.05) between values before and after storage.

Vitamin C in the fruits and vegetables was estimated by three different methods (AOAC, DCPIP and DNP), and expressed in mg/100 g fw. With the AOAC method, Vit. C varied from 7.01 (orange) to 75.08 (dill) before storage, and from 3.90 (orange) to 43.43 (pomegranate) after storage. A significant decrease was observed after 14 days storage at 4 °C, with the highest loss of 71.8% seen in tomato. On the other hand, a significant increase of 34.35% was recorded in pomegranate. The content of Vit. C estimated by the DCPIP method before storage ranged between 9.85(orange) and 186.16 (onions), but after storage, it was between 8.25 (tomato) and 166.88 (onions). Minor changes were observed after storage, with the only significant loss of 31.97% recorded in spinach. The DNP method showed Vit. C contents between 39.03 (orange) and 434.29 (pomegranate) before storage, and between 92.66 (orange) and 401.53 (cauliflower) after storage. Cold storage significantly decreased Vit. C levels in the fruits and vegetables, with a 49.42% loss recorded in sugar beet. On the other hand, a non-significant increase was obtained in cabbage, cauliflower, sapota and orange. Among the three methods used, the highest levels of Vit. C were measured by the DNP method, followed by the DCPIP and AOAC methods (Figure 3).

### 3.4. Anthocyanins

**Figure 4:**
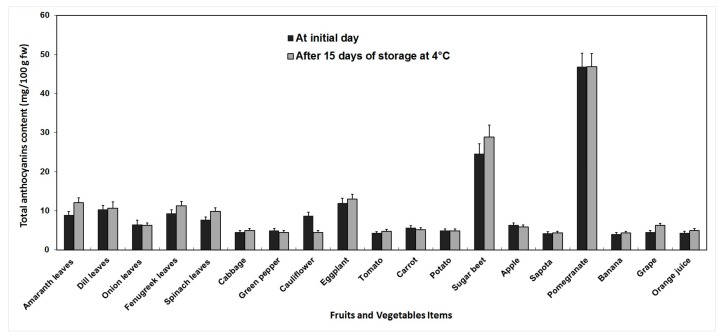
Changes in total anthocyanins content of fruits and vegetables before and after 15 days of storage at 4 °C. Anthocyanins were extracted in acidified methanol and quantified spectrophotometrically using the molar extinction coefficient and molecular weight of the main anthocyanins. Error bars represent standard error of means of three replicates. * indicate significant differences (Fisher’s LSD, *p* < 0.05) between values before and after storage.

Before storage, the TA content of fruits and vegetables varied from 4.05 to 46.78 mg/100 g fw, and after storage it was between 4.34 and 46.90 mg/100 g fw. In the two treatments, banana and pomegranate showed the lowest and the highest TA, respectively. During storage, an increase of TA content was observed, a considerable loss was recorded in cauliflower, but in general, low-temperature storage did not significantly influence TA (Figure 4).

### 3.5. Antioxidant Activity

**Figure 5:**
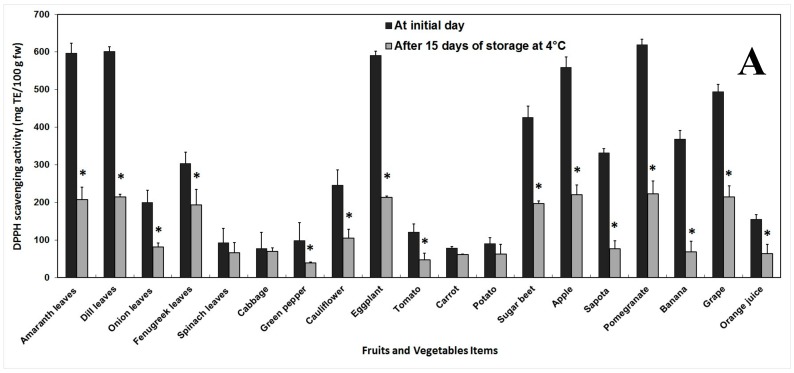

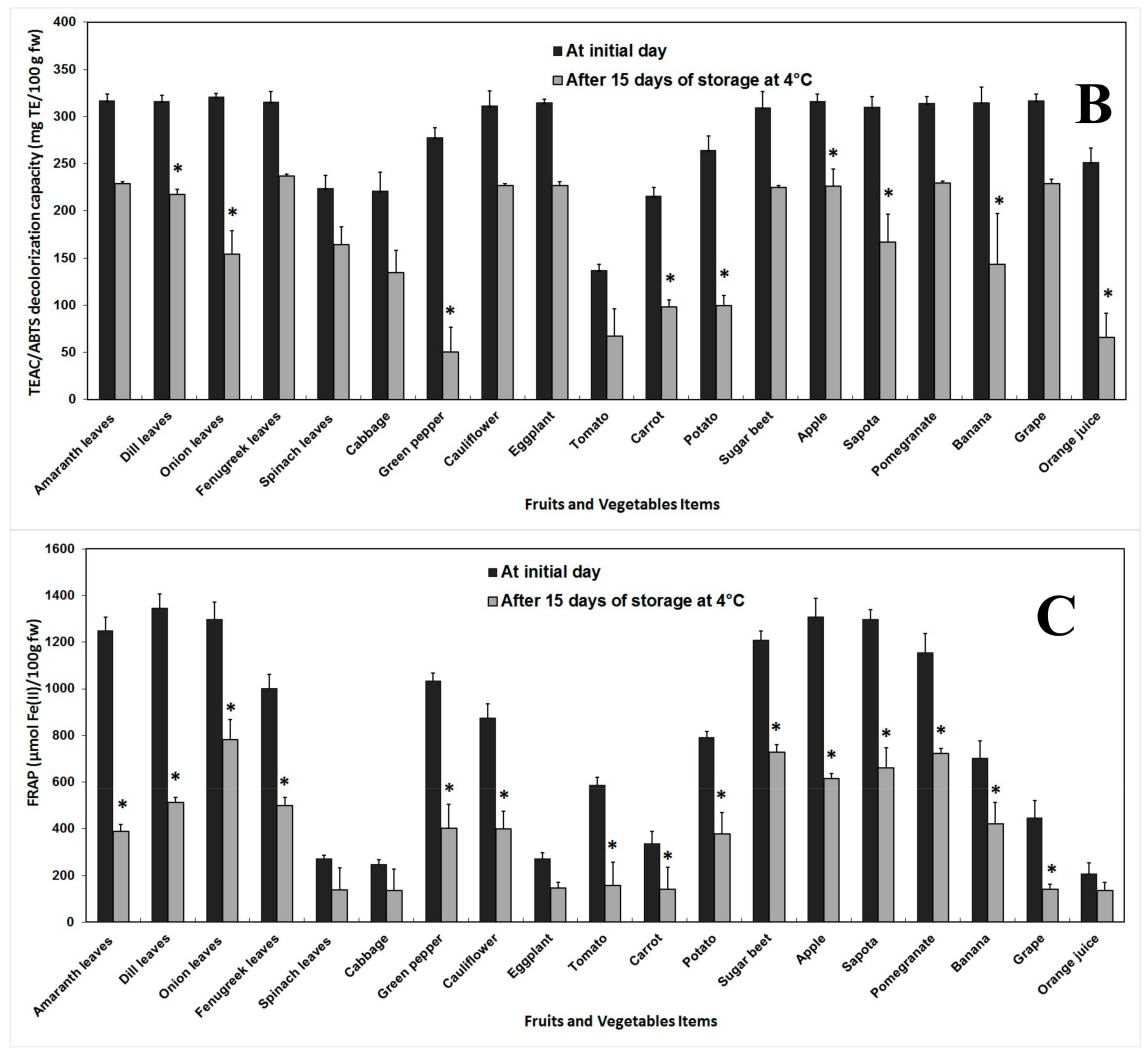
Changes in antioxidant activity of fruits and vegetables phenolic extracts before and after 15 days of storage at 4 °C, evaluated by DPPH scavenging assay (**A**), ABTS decolorization assay (**B**) and FRAP assay (**C**). Antioxidant activity from total phenolic methanol extract was measured with Trolox or ferrous sulfate as standard. Error bars represent standard error of means of three replicates. * indicate significant differences (Fisher’s LSD, *p* < 0.05) between values before and after storage.

The AA was evaluated by the DPPH scavenging assay, ABTS decolorization assay and FRAP assay. Before storage, the DPPH values ranged between 77.22 (cabbage) and 619.40 mg TE/100 g fw (pomegranate). A drastic and significant decrease of AA was observed after storage and it varied from 39.37 (green pepper) to 223.03 TE/100 g fw (pomegranate). The highest loss of activity (81.48%) was recorded with banana, while cabbage exhibited the lowest loss of 10.28% (Figure 5A).

The value of ABTS decolorization was measured in the range of 136.64 (tomato) to 320.87 TE/100 g fw (onion leaves) before storage, and it significantly dropped to the range of 50.76 (green pepper) to 236.65 TE/100 g fw (fenugreek) after storage. An important loss of 81.70% was obtained in green pepper, whereas fenugreek lost only 24.94% of its initial ABTS decolorization power (Figure 5B). The initial estimate of FRAP value was between 207.14 (orange) and 1347.30 µmol Fe(II)/100 g fw (dill). Low-temperature storage induced a significant decrease of FRAP value, and it was situated between 136.85 (orange) and 782.66 µmol Fe(II)/100 g fw (onion) after storage. The highest loss of FRAP was obtained from tomato (73.46%) and orange showed the lowest loss of 33.89% (Figure 5C).

### 3.6. Correlation Analysis

Spearman’s correlation coefficients between phytochemical content and antioxidant parameters are summarized in Table 2. Significant (*p* < 0.05) and highly significant (*p* < 0.01) positive correlations were found between many parameters. The highest correlation between TP and PA were obtained with chlorogenic acid (ρ = 0.423), syringic acid (ρ = 0.403) and sinapic acid (ρ = 0.452).

This suggests that among the 12 identified PAs, these three are the highest contributors of TP in fruits and vegetables, and the changes in TP content could result mainly from the changes in the content of chlorogenic, syringic and sinapic acids. Among the PA, a highly significant high correlation (ρ = 0.836) exist between chlorogenic acid and caffeic acid, which demonstrate similar effect of low temperature on the synthesis and degradation pathways of these PA. Similar observation can be concluded between ferulic and ellagic acid (ρ = 0.803). A highly significant moderate correlation (ρ = 0.661) was found between TP and TA, indicating that anthocyanins account among the most important phenolic compounds in fruits and vegetables.

### 3.7. Principal Component Analysis

**Figure 6:**
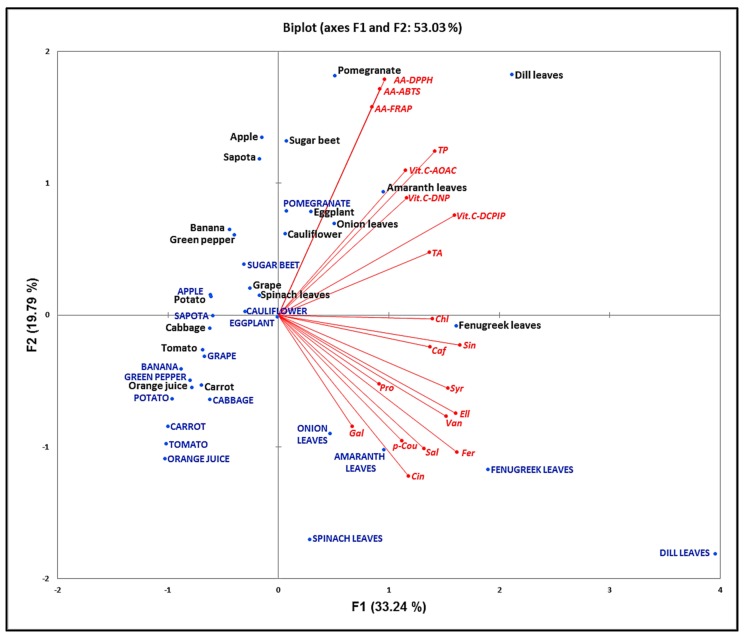
Principal component analysis of phytochemical content and antioxidant activity (red color) of fruits and vegetables before (black color) and after (blue color) storage during 15 days at 4 °C. TP = Total phenolics, Gal = Gallic acid, Pro = protocatechuic acid, Chl = chlorogenic acid, Caf = caffeic acid, Van = vanillic acid, Syr = syringic acid, p-Cou = para-coumaric acid, Fer = ferulic acid, Sin = sinapic acid, Sal = salicylic acid, Ell = ellagic, Cin = *trans*-cinnamic acid, Vit. C-DNP = Vitamin C estimated by DNP method, Vit. C-AOAC = Vitamin C estimated by AOAC method, Vit. C-DCPIP = Vitamin C estimated by DCPIP method, TA = Total anthocyanins, AA-DPPH = Antioxidant activity measured with DPPH, AA-ABTS = Antioxidant activity measured with ABTS, AA-FRAP = Antioxidant activity measured by FRAP.

The PCA biplot (Figure 6) illustrates the relationship between the parameters evaluated in this study, and how they are influenced during low-temperature storage. The biplot clearly showed (red color) a cluster of the three antioxidant analysis methods together, as evidenced by their Spearman’s correlation coefficients. The second cluster contained the antioxidant compounds TP, TA and Vit. C with the three methods of Vit. C estimation grouped together, and the third cluster grouped the 12 PAs, according to the significant correlations found between the respective groups of parameters.

Based on this biplot, it is possible to confirm that before storage (black color), pomegranate and dill located in the top right corner had the highest overall AA, whereas orange juice and carrot, located in the opposite position, showed the lowest AA. Similarly, when considering the three estimation methods of Vit. C or TP content, dill leaves and orange juiced contained the highest and the lowest levels of Vit. C, respectively. It also appeared that fenugreek was the richest in PA.

## 4. Discussion

### 4.1. Total Phenolics

The results of TP content agree with the findings of previous authors, who found similar amounts in fruits and vegetables. Beh et al. [32] found that apple juice had the lowest TP content (5.85 mg GAE/g) while it was 13.19 mg GAE/g for orange juice. Deng et al. [24] showed that the TP content of 56 Chinese vegetables were in the range of 4.99–23.27 mg GAE/g fw. Nour et al. [33] reported lower TP content (418.09 mg GAE/g) in dill leaves. The TP content can be influenced by many factors like genotype, harvest time, growing location and method of extraction, as vigorous extraction methods can lead to an increase in the subsequent measured phenolic content [6,34]. On the other hand, fluctuations of the TP content of fresh fruits and vegetables during storage at 4 °C was observed by many authors [6,7,8,11,35]. The increased synthesis of phenolic compounds under low temperature stress is a response of the plants against adverse climate conditions including chilling injury by synthesizing polyphenolic phytoalexins, through an increase of phenylalanine ammonialyase activity, coupled with low level of polyphenoloxidase activity, that may reduce the oxidation of phenolic substrates to quinones [36,37,38,39]. Oppositely, the decreasing trend also observed may be due to degradation of the polyphenolic compounds, following a change in the pattern of activity of these enzymes. The level of enzyme activity itself depends on the fruit or vegetable matrix, which may have a certain protective effect on the enzyme when submitted to low temperature [40].

### 4.2. Phenolic Acids

Except for protocatechuic acid, all the other 11 PAs detected in this study were also reported in dill leaves [33]. These authors also found that sinapic and vanillic acids were prevalent in dill but in this study, we rather found sinapic and ellagic acids. Moreover, chlorogenic acid and gallic acid were the most detected in the vegetables studied by Deng et al. [24], which doesn’t totally agree with our findings. The dissimilarities can be due to the differences in species, growing conditions, storage duration and condition, as well as the method used for the extraction and estimation of the PA. The decrease in PA content during low-temperature storage can be attributed to enhancement of the degradation of polyphenols through storage conditions, by an active enzyme system in the food tissue including anthocyanases, polyphenol oxidase and peroxidase. On the other hand, the increase of chlorogenic acid during cold storage can be attributed to the accumulation of sugars, which act as substrates for its synthesis [41]. Additionally, accumulation of the degradation products of chlorogenic acid, isochlorogenic acid and acylated anthocyanins present in dill can justify the observed drastic increase of caffeic acid content [42]. Galani et al. [8] suggested that the observed changes of PA during storage in a species-dependent pattern can be explained by the fact that genes and enzymes governing the synthesis and the degradation of PA in each fruit and vegetable respond to low temperature condition differently in each food item, i.e., in a species-dependent way.

### 4.3. Vitamin C

Several other findings support our results. The Indian fruits and vegetables contained the following amounts of Vit. C (mg/100 g fw) measured by the AOAC method: banana (18.65), sapota (34.49), pomegranate (34.14), carrot (29.92), cabbage (35.55), cauliflower (39.42) and potato (31.32) [20]. Adebayo [21] reported Vit. C contents of bananas extracted by orthophosphoric acid, ranging from 0.3 mg/100 g (AOAC method) to 19.0 mg/100 g (DNP method). The Vit. C content measured by the DNP method varied between 40.50 and 55.97 mg/100 g fw in different varieties of tomato and it was 63.63 mg/100 g fw in Valencia orange [19]. High ascorbic acid content (204.55 mg/100 g fw) measured by HPLC was found in dill and after 12 days storage at 4 °C in the refrigerator, it dropped by 8% [16]. Similar observations were reported by Howard et al. [13] and Lisiewska et al. [14].

In addition to the species, varieties, harvest time and postharvest handling which can affect Vit. C content, the extraction solvent and the method of measurement also have a significant impact. Depending on the extraction solvent, the total vitamin C of ripe orange was between 66 to 77 mg/100 g fw and for ripe banana, it ranged between 1.57 and 6.61 mg/100 g fw, for 0.1% oxalic acid and 3% metaphosphoric acid-8% acetic acid, respectively [18]. The range of ascorbic acid content of 5 selected Nigerian fruits was lower with titrimetric method 0.20 to 13.20 mg/100 g) than with spectrophotometric method (7.76 to 87.1 mg/100 g) [21]. The DCPIP titrimetric method is applicable to the determination of reduced form of ascorbic acid (L-ascorbic acid) only, and not for the total vitamin C, i.e., ascorbic acid plus dehydroascorbic acid, both detected by the spectrophotometric methods DCPIP and DNP [19].

Vitamin losses during handling and storage are due to their water solubility, thermic degradation and enzymatic oxidation. The presence of chelating agents or ascorbic acid oxidase inhibitors in a food matrix can protect vitamin C against oxidation [43], and may justify the various patterns of changes observed in different fruits and vegetables. Additionally, the changes in Vit. C content can result from alterations in expression of genes and activity of enzymes in the pathway of Vit. C metabolism during storage. Vitamin C might be synthesized as a response to the stress caused by the storage temperature and then used as antioxidant compound in response to oxidative stress caused by low-temperature storage. The level of expression of these genes and the activity of the subsequent enzymes could be temperature-dependent [8].

### 4.4. Anthocyanins

Our findings on TA content of fruits and vegetables coincide with the results reported in small fruits [11] and [43] and in potato [3], but Burgos et al. [26] found higher amounts. Increases of TA during storage of fruits and vegetables in cold conditions was previously observed [11,43]. The authors suggested that the release of membrane bound anthocyanins enhanced by cold condition can justify the higher TA content obtained after cold storage. Moreover, loss of anthocyanins can be due to their degradation in plant tissues by enzyme systems such as glycosidases (anthocyanases), polyphenoloxidases and peroxidases [42].

### 4.5. Antioxidant Activity

Our DPPH radical-scavenging activity results confirm other findings [11], but Deng et al. [24] found lower values of ABTS and FRAP, while Nour et al. [33] obtained lower values of DPPH than ours. Also, [32] found that DPPH radical-scavenging activity of apple juice was lower than orange antioxidant activity, which did not agree with our results. Our findings demonstrate a decrease of AA during storage of fruits and vegetables, different effect of cold storage on AA of fruits and vegetables have been reported by various authors: stability of AA during postharvest storage was observed in apricots, plums and grapes [35] and in tomatoes [44]; increase in AA during storage was shown during refrigerated storage of celery [45] and small fruits [11]. As observed in this study, the antioxidant capacity varies with the assay used for its estimation, and we found that highest values were obtained with FRAP assay, followed by DPPH assay, and ABTS assay showed the lowest AA values. Among different spices, the highest AA measured by the DPPH assay was observed in rosemary, whereas oregano had the highest activity in the ORAC test, and parsley showed the lowest AA in both of the assays [46]; antioxidant capacity of 50 most popular antioxidant-rich fruits, vegetables and beverages in the US diet detected by ABTS assay was significantly higher compared to DPPH assay [30]. Antioxidant activity varies with the species, the method of evaluation, and the extraction solvent [24]. Decrease of AA during storage can be attributed to a decreased level of total phenolics, phenolic acids vitamin C and other compounds like anthocyanins, carotenoids and flavonoids when the fruits and vegetables are stored [8].

### 4.6. Correlation Analysis

It was assumed that strong correlations would be found between the values of Vit. C estimated with the three methods. However, our data do not agree with this assumption since the AOAC method data only moderately correlated with the DNP method (ρ = 0.650) and DCPIP spectrophotometric method (ρ = 0.535) results, while a low correlation was found between DNP and DCPIP (ρ = 0.469). This demonstrates the necessity of combining two or more Vit. C estimation methods in order to get a better view of the effective Vit. C content in food items [18,19,20,21].

Antioxidant activity mostly correlated with TP: the correlation was high with DPPH (ρ = 0.808), moderate with ABTS (ρ = 0.690) and low with FRAP (ρ = 0.458). Deng et al. [24] demonstrated that TP showed a strong correlation with total antioxidant capacities (r = 0.857 and 0.810 for FRAP and ABTS, respectively), which indicated that phenolic compounds could be one of the main contributors to the antioxidant capacities of vegetables. Besides, no any significant correlation was shown between AA and PA, suggesting a synergy among the PA to exert their AA potential. Considering all the estimation methods used, low to moderate correlations existed between AA and Vit. C content. In fact, non-phenolic antioxidants like Vit. C, carotenoids and minerals significantly contribute to AA [8,11], but Vit. C may not be the most important contributor of AA potential of fruits and vegetables [22,23]. Total anthocyanins negligibly and non-significantly correlated with FRAP, but a low correlation was found between TA and DPPH (ρ = 0.429) or ABTS (ρ = 0.482). This shows that, although they contribute to AA potential, anthocyanins found in fruits and vegetables are not the key-compounds responsible of their antioxidant properties; it also implies that these anthocyanins are more involved in radical-scavenging activity (DPPH and ABTS), than in ferric reducing antioxidant power.

Between the methods for AA evaluation, high positive correlation was found between DPPH and ABTS (ρ = 0.867), whereas FRAP moderately correlated with DPPH (ρ = 0.613) and ABTS (ρ = 0.660). Many previous reports support close correlation results between DPPH and ABTS for evaluation of AA [6,7,8,25,30]. There is a high variability among the various methods of AA evaluation, but DPPH and ABTS remain the more accurate and less prone to variability [47,48]. Faller and Fialho [49] also reported positive correlations between polyphenols and AA in raw and cooked potatoes, carrots, onions, broccoli, and white cabbage. Floegel et al. [30] found that antioxidant capacity detected by ABTS assay was stronger positively associated to the other parameters.

### 4.7. Principal Component Analysis

The PCA biplot (Figure 6) illustrates the relationship between the parameters evaluated in this study, and how they are influenced during low-temperature storage. The biplot clearly showed (red color) a cluster of the three antioxidant analysis methods together, as evidenced by their Spearman’s correlation coefficients. The second cluster contained the antioxidant compounds TP, TA and Vit. C with the three methods of Vit. C estimation grouped together, and the third cluster grouped the 12 PAs, according to the significant correlations found between the respective groups of parameters. Based on this biplot, it is possible to confirm that before storage (black color), pomegranate and dill located in the top right corner had the highest overall AA, whereas orange juice and carrot, located in the opposite position, showed the lowest AA. Similarly, when considering the three estimation methods of Vit. C or TP content, dill leaves and orange juiced contained the highest and the lowest levels of Vit. C, respectively. It also appeared that fenugreek was the richest in PA.

The positions of variables after storage (blue color) show the influence of cold storage on antioxidant parameters. Dill was shifted to the lower right corner of the plot, indicating a decrease in AA, Vit. C and TP, but an increase in PA. Comparable observation can be seen with fenugreek. With pomegranate, sugar beet and eggplant however, the decrease of AA, Vit. C and TP was followed by little or no change in PA. In general, PCA revealed that cold storage caused a shift in the AA and phytochemical content of the fruits and vegetables, the pattern of shifting varied with the food item considered, and differed from one parameter to the other: while TP, Vit. C, PA and AA decreased with storage, PA rather increased. The PCA results confirm the correlation analysis and demonstrate the usefulness of this multivariate analysis in the classification of fruits and vegetables on the basis of their AA and individual antioxidant compounds [31,46].

## 5. Conclusions

This study has demonstrated that when fruits and vegetables are stored in refrigerator for 15 days, their content in TP, Vit. C and TA decrease while PA rather increase. Subsequently, their AA also decreases, suggesting a key-role of TP, followed by Vit. C and TA in antioxidant capacity. High amounts of phytochemicals and strong antioxidant capacities were found in dill, pomegranate and amaranth, which could be interesting dietary sources of natural antioxidants for prevention of diseases caused by oxidative stress. Increase of PA during storage in refrigerator suggest the use of cold storage as biofactories for the production of PA-rich foods, the stressed fruits and vegetables with increased PA could be used as raw material for extraction of high value antioxidant PA with potential applications in the pharmaceutical, nutraceuticals and functional foods industries. Further studies on effect of cooking and bioavailability of these antioxidant compounds will provide more information to consumers and nutritionists, while investigating the role of enzymes and genes of phenylpropanoid pathway during storage will help breeders in selection and development of more health-promoting fruits and vegetables.

## Figures and Tables

**Table 1:** Phenolic acid composition of fruits and vegetables before and after 15 days of storage at 4 °C.

Fruits and Vegetables	Phenolic Acids Content (mg/100 g fw)											
	Gal	Pro	Chl	Caf	Van	Syr	p-Cou	Fer	Sin	Sal	Ell	Cin
**Amaranth leaves**	7960.53 ± 7.95	121.44 ± 0.12	175.01 ± 0.03	3.57 ± 0.19	27.15 ± 4.90	0.68 ± 0.01	0.67 ± 0.00	2.15 ± 0.06	384.57 ± 0.25	5.97 ± 0.03	188.74 ± 3.33	82.73 ± 0.12
	(18,886.11 ± 13.34) *	(335.09 ± 0.55) *	(236.67 ± 0.51)	(3.20 ± 0.04)	(160.82 ± 1.20)	(1.35 ± 0.01)	(1.41 ± 0.00) *	(2.22 ± 0.01)	(361.87 ± 2.27)	(7.80 ± 0.09)	(636.05 ± 9.22)	(52.35 ± 0.31)
**Dill leaves**	-	-	236.23 ± 0.00	1.16 ± 0.00	432.64 ± 0.00	17.25 ± 0.00	-	1.76 ± 0.02	2587.19 ± 0.52	7.97 ± 0.08	12,231.48 ± 0.68	24.01 ± 0.25
	(648.90 ± 7.93)	-	(912.15 ± 0.79) *	(57.72 ± 0.25) *	(1714.27 ± 0.66) *	(52.31 ± 0.15) *	(2.28 ± 0.01) *	(5.61 ± 0.07) *	(5001.95 ± 6.4) *	(42.21 ± 0.89) *	(12,429.06 ± 5.50)	(173.46 ± 0.28) *
**Onion leaves**	-	-	66.94 ± 0.05	1.25 ± 0.01	210.41 ± 0.60	1.73 ± 0.00	0.34 ± 0.00	1.01 ± 0.02	1392.04 ± 0.13	11.49 ± 0.16	3655.16 ± 3.75	-
	(36.54 ± 1.42)	(23.66 ± 0.21)	(74.58 ± 0.05)	(3.94 ± 0.02)	(856.61 ± 0.06) *	(3.28 ± 0.14)	(0.71 ± 0.02) *	(7.60 ± 0.01) *	(1068.49 ± 1.34)	(9.90 ± 0.15)	(2187.94 ± 1.99)	(19.72 ± 0.02)
**Fenugreek leaves**	-	26.45 ± 0.34	35.8 ± 0.02	3.18 ± 0.03	1530.38 ± 0.92	36.39 ± 0.01	2.68 ± 0.00	6.98 ± 0.02	870.21 ± 1.21	11.37 ± 0.03	681.97 ± 2.30	18.86 ± 0.25
	-	(10.87 ± 0.47)	(56.88 ± 0.01)	(4.96 ± 0.06)	(2708.97 ± 0.38) *	(52.73 ± 0.02) *	(1.06 ± 0.02) *	(10.52 ± 0.19) *	(1374.82 ± 0.00)	(27.22 ± 0.31) *	(989.15 ± 5.85)	(17.54 ± 1.38)
**Spinach leaves**	-	-	0.16 ± 0.01	0.31 ± 0.03	206.69 ± 0.00	0.38 ± 0.00	0.14 ± 0.00	0.35 ± 0.00	105.49 ± 0.01	1.17 ± 0.02	323.05 ± 5.06	48.24 ± 0.48
	-	-	(1.21 ± 0.01)	-	(413.8 ± 0.31)	(1.40 ± 0.01)	(2.21 ± 0.01) *	(1.77 ± 0.00)	(177.33 ± 0.6)	(5.35 ± 0.05)	(2616.59 ± 9.61) *	(306.8 ± 0.81) *
**Cabbage**	-	-	1.45 ± 0.00	0.05 ± 0.00	244.24 ± 2.35	0.5 ± 0.00	0.05 ± 0.00	0.42 ± 0.01	8.18 ± 0.06	0.31 ± 0.01	630.94 ± 1.8	3.23 ± 0.02
	(11.44 ± 0.06)	(1.22 ± 0.03)	(3.26 ± 0.08)	(0.22 ± 0.00)	(197.15 ± 0.15)	(0.88 ± 0.02)	(0.19 ± 0.00)	(2.01 ± 0.01) *	(14.12 ± 0.21)	(4.29 ± 0.03)	(1046.28 ± 0.99)	(5.31 ± 0.01)
**Green pepper**	-	8.31 ± 0.02	3.49 ± 0.03	0.09 ± 0.00	1.63 ± 0.04	0.03 ± 0.00	0.02 ± 0.00	0.02 ± 0.00	4.10 ± 0.20	0.31 ± 0.06	7.31 ± 0.16	3.63 ± 0.00
	(3.40 ± 0.61)	(19.36 ± 0.01)	(1.02 ± 0.04)	-	(8.53 ± 0.08)	(0.05 ± 0.00)	(0.02 ± 0.01)	(0.1 ± 0.01)	(17.77 ± 0.02)	(0.35 ± 0.05)	(96.10 ± 0.42)	(2.78 ± 0.00)
**Cauliflower**	-	30.83 ± 0.22	13.21 ± 0.02	3.89 ± 0.02	410.92 ± 0.07	0.97 ± 0.00	0.70 ± 0.00	0.56 ± 0.00	6.04 ± 0.11	3.21 ± 0.05	611.03 ± 0.29	4.26 ± 0.04
	-	(18.69 ± 0.30)	(7.49 ± 0.01)	(0.29 ± 0.00)	(221.11 ± 0.12)	(0.32 ± 0.00)	(0.53 ± 0.04)	(1.55 ± 0.01)	-	(1.38 ± 0.04)	(472.51 ± 1.73)	-
**Eggplant**	-	-	638.03 ± 0.03	27.91 ± 0.02	-	3.09 ± 0.07	-	-	436.38 ± 0.07	-	-	-
	-	-	(611.38 ± 0.24)	(30.93 ± 0.03)	-	(3.88 ± 0.01)	-	-	(78.97 ± 0.27)	-	-	-
**Tomato**	-	-	20.09 ± 3.14	1.96 ± 0.00	24.18 ± 0.06	-	-	0.70 ± 0.03	114.05 ± 0.09	6.48 ± 0.01	51.84 ± 0.15	2.84 ± 0.02
	(26.18 ± 0.22)	-	(24.15 ± 0.01)	(1.34 ± 0.02)	-	-	-	(0.70 ± 0.01)	(48.84 ± 0.18)	(6.09 ± 0.03)	(308.61 ± 2.36)	-
**Carrot**	-	-	36.71 ± 1.14	1.65 ± 0.02	265.02 ± 9.41	1.65 ± 0.26	0.32 ± 0.05	0.17 ± 0.08	48.52 ± 3.66	3.80 ± 0.41	-	32.31 ± 4.82
	-	-	(20.26 ± 0.62)	(1.49 ± 0.02)	(9.95 ± 1.60)	(0.44 ± 0.07)	(0.24 ± 0.04)	(0.15 ± 0.04)	(3.13 ± 7.32)	(2.50 ± 0.56)	(53.32 ± 4.16)	(5.65 ± 0.90)
**Potato**	-	-	11.45 ± 0.04	1.01 ± 0.02	6.40 ± 0.01	0.33 ± 0.00	0.27 ± 0.01	0.12 ± 0.02	8.03 ± 0.07	1.08 ± 0.04	46.28 ± 0.24	9.54 ± 0.14
	-	-	(18.13 ± 0.04)	(1.18 ± 0.00)	(10.23 ± 0.07)	(0.65 ± 0.02)	(0.03 ± 0.00)	(0.11 ± 0.01)	(5.92 ± 0.01)	(1.37 ± 0.01)	(41.48 ± 0.22)	(6.25 ± 0.02)
**Sugar beet**	-	-	7.48 ± 0.12	-	-	1.31 ± 0.00	-	0.54 ± 0.02	46.21 ± 0.09	-	274.08 ± 0.79	-
	(10.08 ± 2.97)	-	-	(0.34 ± 0.02)	-	(0.56 ± 0.01)	-	-	(72.67 ± 0.22)	(3.57 ± 0.12)	(117.13 ± 0.73)	(3.62 ± 0.01)
**Apple**	-	-	1.11 ± 0.01	0.35 ± 0.06	31 ± 0.07	0.21 ± 0.00	-	-	34.34 ± 0.09	0.57 ± 0.03	-	-
	-	-	(33.23 ± 0.03)	(0.95 ± 0.02)	(34.15 ± 0.05)	(0.27 ± 0.01)	-	(0.18 ± 0.00)	(41.62 ± 0.17)	(0.75 ± 0.01)	-	-
**Sapota**	-	-	16.29 ± 0.25	0.23 ± 0.00	21.4 ± 0.02	0.07 ± 0.00	-	-	6.82 ± 0.05	0.22 ± 0.01	-	-
	-	(2.17 ± 0.02)	(7.36 ± 0.01)	(0.22 ± 0.05)	(15.05 ± 0.07)	(0.06 ± 0.02)	-	-	(26.45 ± 0.04)	(1.12 ± 0.01)	(35.02 ± 0.97)	-
**Pomegranate**	-	-	3.04 ± 0.64	0.13 ± 0.00	68.47 ± 0.14	0.16 ± 0.00	0.18 ± 0.01	-	38.33 ± 0.06	1.04 ± 0.01	40.36 ± 5.35	2.16 ± 0.04
	-	-	(3.38 ± 0.01)	(0.15 ± 0.07)	(14.62 ± 0.01)	(0.13 ± 0.00)	(0.11 ± 0.03)	(0.12 ± 0.02)	(5.23 ± 0.11)	(2.75 ± 0.01)	(33.68 ± 0.22)	-
**Banana**	-	-	0.51 ± 0.00	-	5.69 ± 0.00	-	0.39 ± 0.00	0.09 ± 0.00	-	-	-	-
	-	-	-	-	-	-	(0.20 ± 0.01)	-	-	-	-	-
**Grape**	-	-	8.18 ± 0.02	-	-	-	0.18 ± 0.00	-	94.74 ± 0.01	27.81 ± 0.02	79.01 ± 0.87	2.97 ± 0.02
	(15.95 ± 0.05)	-	(5.41 ± 0.01)	(0.13 ± 0.01)	(8.91 ± 0.16)	(0.09 ± 0.02)	(0.11 ± 0.01)	-	(42.08 ± 0.01)	(13.95 ± 0.01) *	-	-
**Orange juice**	-	20.44 ± 0.11	-	-	89.97 ± 0.61	3.77 ± 0.01	0.75 ± 0.01	-	70.03 ± 0.16	1.06 ± 0.02	-	25.03 ± 0.06
	-	-	-	-	(42.78 ± 0.04)	(2.65 ± 0.04)	(0.09 ± 0.03) *	(0.21 ± 0.00)	(30.76 ± 0.11)	(7.24 ± 0.01) *	-	(19.22 ± 0.05)

Chromatograms were integrated using Empower 3 software, peaks were identified by comparing with retention times and UV spectra of standards, and each phenolic acid content was calculated according to peak area. Values are presented as the mean ± SE of three replicates. Values in brackets represent the phenolic acid content after 15 days of storage at 4 °C. * indicate significant differences (Fisher’s LSD, *p* < 0.05) between values before and after storage. Gal = Gallic acid, Pro = protocatechuic acid, Chl = chlorogenic acid, Caf = caffeic acid, Van = vanillic acid, Syr = syringic acid, p-Cou = para-coumaric acid, Fer = ferulic acid, Sin = sinapic acid, Sal = salicylic acid, Ell = ellagic acid and Cin = *trans*-cinnamic acid.

**Table 2:** Spearman’s correlation coefficients between phytochemical content and antioxidant parameters of fruits and vegetables before and after 15 days of storage at 4 °C.

	TP	Gal	Pro	Chl	Caf	Van	Syr	p-Cou	Fer	Sin	Sal	Ell	Cin	Vit. C-DNP	Vit. C-AOAC	Vit. C-DCPIP	TA	AA-DPPH	AA-ABTS
Gal	0.054																		
Pro	0.110	0.346 *																	
Chl	0.423 **	0.220	0.137																
Caf	0.324 *	0.259	0.202	0.836 **															
Van	0.087	0.029	0.356 *	0.235	0.308														
Syr	0.403 *	0.064	0.229	0.458 **	0.497 **	0.584 **													
p-Cou	0.109	0.191	0.486 **	0.114	0.154	0.588 **	0.400 *												
Fer	0.126	0.332 *	0.398 *	0.446 **	0.426 **	0.690 **	0.489 **	0.526 **											
Sin	0.452 **	0.311	0.092	0.549 **	0.499 **	0.393 *	0.610 **	0.187	0.452 **										
Sal	0.068	0.413 *	0.193 *	0.362 *	0.342 *	0.510 **	0.335 *	0.454 **	0.589 **	0.585 **									
Ell	0.171	0.342 *	0.313	0.311	0.291	0.583 **	0.412 *	0.433 **	0.803 **	0.482 **	0.579 **								
Cin	0.134	0.261	0.330 *	0.146	0.233	0.577 **	0.546 **	0.586 **	0.533 **	0.426 **	0.487 **	0.468 **							
Vit. C-DNP	0.412 *	0.011	0.341 *	0.127	0.052	0.361 *	0.078	0.150	0.304	0.056	0.093	0.453 **	0.006						
Vit. C-AOAC	0.396 *	−0.066	0.106	0.101	0.179	0.351 *	0.029	0.049	0.304 *	0.174	0.053	0.384 *	0.034	0.650 **					
Vit. C-DCPIP	0.525 **	0.098	0.184	0.358 *	0.319	0.383 *	0.383 *	0.177	0.299	0.541 **	0.204	0.417 *	0.157	0.469 **	0.535 **				
TA	0.661 **	0.175	−0.012	0.335 *	0.398 *	0.173	0.548 **	0.087	0.237	0.493 **	0.216	0.306	0.197	0.267	0.194	0.563 **			
AA-DPPH	0.808 **	−0.097	−0.034	0.277	0.168	0.014	0.121	−0.057	−0.108	0.299	−0.019	−0.077	−0.174	0.446 **	0.484 **	0.440 **	0.429 **		
AA-ABTS	0.690 **	−0.237	0.076	0.208	0.081	0.028	0.117	0.093	−0.105	0.234	−0.020	−0.014	−0.138	0.434 **	0.528 **	0.419 **	0.482 *	0.867 **	
AA-FRAP	0.458 **	−0.120	0.109	0.166	0.119	0.072	−0.069	−0.100	0.029	0.103	−0.051	0.115	−0.150	0.519 **	0.650 **	0.567 **	0.176	0.613 **	0.660 **

TP = Total phenolics, Gal = Gallic acid, Pro = protocatechuic acid, Chl = chlorogenic acid, Caf = caffeic acid, Van = vanillic acid, Syr = syringic acid, p-Cou = para-coumaric acid, Fer = ferulic acid, Sin = sinapic acid, Sal = salicylic acid, Ell = ellagic, Cin = *trans*-cinnamic acid, Vit. C-DNP = Vitamin C estimated by DNP method, Vit. C-AOAC = Vitamin C estimated by AOAC method, Vit. C-DCPIP = Vitamin C estimated by DCPIP method, TA = Total anthocyanins, AA = Antioxidant.
